# Molecular Characterization, Evolutionary Analysis, and Expression Profiling of *BOR* Genes in Important Cereals

**DOI:** 10.3390/plants11070911

**Published:** 2022-03-29

**Authors:** Himanshu Sharma, Alok Sharma, Ruchika Rajput, Sukhjeet Sidhu, Harpal Dhillon, Praveen Chandra Verma, Ashutosh Pandey, Santosh Kumar Upadhyay

**Affiliations:** 1 Department of Botany, Panjab University, Chandigarh 160014, India; sharma.himanshu469@gmail.com (H.S.); sharmaalok0001@gmail.com (A.S.); 2 Department of Bio-Technology, I.K. Gujral Punjab Technical University, Kapurthala 144603, India; 3 National Institute of Plant Genome Research, Aruna Asaf Ali Marg, New Delhi 110067, India; ruchikarajput72@gmail.com (R.R.); ashutosh@nipgr.ac.in (A.P.); 4 Department of Biotechnology, SUSCET, Tangori, Mohali 140306, India; s_sukhjeetk@yahoo.com; 5 Centre for Infectious Disease and Vector Research, Department of Nematology, Institute for Integrative Genome Biology, University of California Riverside, Riverside, CA 92521, USA; harpald@ucr.edu; 6 Plant Molecular Biology and Genetic Engineering Department, CSIR-National Botanical Research Institute, Council of Scientific and Industrial Research, Lucknow 226001, India; praveencverma@nbri.res.in; 7 Academy of Scientific and Innovative Research (AcSIR), Ghaziabad 201002, India

**Keywords:** BOR, boron, *T. aestivum*, stress, qRT-PCR

## Abstract

Boron (B) is an essential micronutrient of plants. Plants grapple with a narrow range of B between its toxicity and deficiency. B homeostasis mechanism is required to rescue plants from such a quagmire. B transporters are specialized proteins involved in the homeostasis of B. In the present study, a total of 29 *BOR* genes were identified in five major cereals, including three *BORs* in each *Brachypodium distachyon* and *Sorghum bicolor*, four in *Oryza sativa*, six in *Zea mays*, and 13 in *Triticum aestivum*. Multiple sequence alignments, domain structure analyses, and phylogenetic analysis indicated the conserved nature of the BOR protein family. Duplication events and Ka/Ks analysis of *TaBORs* showed the role of segmental duplication events and purifying selection in the expansion of the BOR family in *T. aestivum*. Furthermore, in silico expression and co-expression analyses under biotic and abiotic stress conditions depicted their involvement in combating such conditions. Moreover, qRT-PCR of *TaBORs* in B treatment suggested the roles of *BOR* genes in B stress management. The present study hints at the conserved nature of BOR proteins and their different aspects. The study will lay down a way for several crop improvement programs.

## 1. Introduction

Boron (B) is an essential nutrient for plants. It is known for its role in stabilizing the molecules with *cis* diol groups [1,2]. B plays a pivotal role in cell wall strengthening, as it binds apiose residues in rhamnogalacturonan (RG-II) to cross-link pectic polysaccharides [2,3,4]. Furthermore, B is involved in multiple biological processes, including nucleic acid metabolism, assimilate partitioning, reproductive growth, and membrane integrity [2,5]. B is generally present in the form of water-soluble molecules in soil and absorbed by plants in the form of boric acid (H_3_BO_3_) and borate (B(OH)_4_^−^) [1,5].

B deficiency, as well as the toxicity of B, can cause various diseases in plants [6,7]. B deficiency is manageable using B fertilizers; however, the toxicity of B is difficult to manage. Thus, a homeostasis mechanism of B is required in plants. Plants have three different mechanisms of B uptake, including passive diffusion, facilitated diffusion with the help of B channels, and active transport using B transporters (BORs) [1]. The majority of B transporters are exporters (effluxers), as B is exported from pericycle or endodermal cells into the xylem vessels for supplying B to the shoots and other parts [1,8]. Moreover, BORs transport B from endodermal cells, where the Casparian strip is present, to the xylem vessels. These are generally polarly localized and transport B in the form of borate (an anionic form of boric acid). The first B transporter, BOR1, was reported in *Arabidopsis thaliana* in 2002 by Takano et al. [9]. A mutant of BOR1, i.e., *A. thaliana* mutant *bor1-1*, when grown on a low B medium, showed a reduction in the size of rosette leaves and seed production, whereas it resumed normal growth after the employment of adequate B [9]. Moreover, ubiquitination-mediated vacuolar trafficking checks the BOR1 activity in order to combat high B conditions [10,11]. Another transporter, AtBOR4, is involved in the tolerance of high B conditions and is important for directional B transport from roots to soil to avoid B overaccumulation [12]. Hitherto, seven *BOR* genes have been reported in *A. thaliana* [13]. Furthermore, a number of *BOR* genes have been reported in different plants, including *Brassica napus*, *Citrus macrophylla*, *Hordeum vulgare*, *Oryza sativa*, *Populus trichocarpa*, *Triticum aestivum*, and *Vitis vinifera* [14,15,16,17,18,19,20,21]. Moreover, multiple *BOR* genes have been found to be involved in plant growth and stress tolerance [22,23]. For instance, the *SiBOR1* gene of foxtail millet is suggested to be involved in cell wall integrity, cellular homeostasis, and panicle development [23]. Another gene, *HvBOR2*, is known for B tolerance properties in barley [15]. However, the majority of these studies are focused on the analyses of some specific *BOR* genes.

In this study, we have identified and characterized the *BOR* gene family in five major cereals, including *Brachypodium distachyon*, *Oryza sativa*, *Sorghum bicolor*, *Triticum aestivum*, and *Zea mays*. These were studied for their characteristic features, such as gene structure, exon-intron architecture, protein structure, physicochemical properties of BOR proteins, and *cis*-regulatory elements. To explore the evolutionary features of the *BOR* gene family, phylogenetic analysis, duplication event (DE) analysis, and Ka/Ks substitutions analysis were performed. Further, expression analysis in each cereal was carried out for various tissues during their developmental stages to unveil the functional characteristics of BOR transporters. Expression studies were also carried out for *T. aestivum BOR* (*TaBOR)* genes during biotic (*Puccinia striiformis* (Pst) and *Blumeria graminis* (Bgt)) and abiotic stress [heat stress (HS), drought stress (DS), combined heat-drought stress (HD), and salt stress] conditions to identify their possible roles in stress response. The qRT-PCR experiments of *TaBOR* genes under B stress were performed to understand their response to B stress. Co-expression analysis was done in each cereal to study the interaction of *BORs* to other transcripts of respective cereals. This study will provide a helping hand to various functional studies and crop improvement programs.

## 2. Results

### 2.1. Identification, Distribution, and Paralogous Analysis of BOR Genes

**Figure 1:**
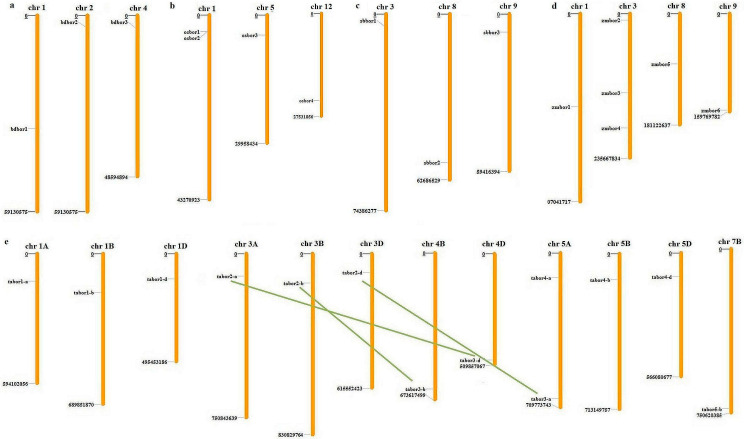
Chromosomal localization and duplication events analyses of *BOR* genes in five cereals. The scattered distribution of *BOR* genes of (**a**) *B. distachyon*, (**b**) *O. sativa*, (**c**) *S. bicolor*, (**d**) *Z. mays*, and (**e**) *T. aestivum* at their respective chromosomes is depicted by the image. The green lines indicate the three segmental duplication events identified in *T. aestivum*.

Genome-wide identification of BORs in the five cereals resulted in a varied number of genes. (Figure 1a–e and Table S1). All the BOR proteins had a highly conserved anion exchanger bicarbonate (HCO_3_^−^) domain. *B. distachyon* consisted of three *BOR* genes (*BdBOR1, BdBOR2* and *BdBOR3)*, which were present on chromosomes 1, 2, and 4, respectively (Figure 1a). In *O. sativa*, four *BOR*s were located on three different chromosomes. *OsBOR1* and *OsBOR2* were present at chromosome 1, while *OsBOR3* and *OsBOR4* were present at chromosomes 5 and 12, respectively (Figure 1b). A total of three *BOR*s were identified in *S. bicolor*. *SbBOR1*, *SbBOR2*, and *SbBOR3* were distributed on the 3rd, 8th, and 9th chromosomes, respectively (Figure 1c). *S*ix *BORs* were identified in *Z. mays*, where *ZmBOR2*, *ZmBOR3*, and *ZmBOR4*, were present on chromosome 3 and *ZmBOR1*, *ZmBOR5*, and *ZmBOR6* were present on chromosomes 1, 8, and 9, respectively (Figure 1d). In the allohexaploid *T. aestivum*, a total of 13 *BOR* genes were identified. These genes were grouped into five different homeologous groups, including *TaBOR1*, *2*, *3*, *4*, and *5* (Figure 1e). Most of the chromosomes possessed only one *BOR* gene each; however, chromosome 5A had two *BOR* genes. No *BOR* could be located on the 2nd and 6th chromosomes of all the subgenomes, on the 7th chromosome of the A and D subgenomes, and the 4th chromosome of the A subgenome. The subgenomic distribution analysis of *TaBORs* suggested nearly equal contribution of each A, B, and D subgenome, with 31%, 31%, and 38%, respectively. The paralogous analysis resulted in the prediction of three DEs in *T. aestivum*. All three DEs were found to be segmental duplication (Figure 1a–e and Table S2). None of the other cereals, including *B. distachyon, O. sativa, S. bicolor*, and *Z. mays*, showed any duplication event.

### 2.2. Multiple Sequence Alignment and Evolutionary Analysis

**Figure 2:**
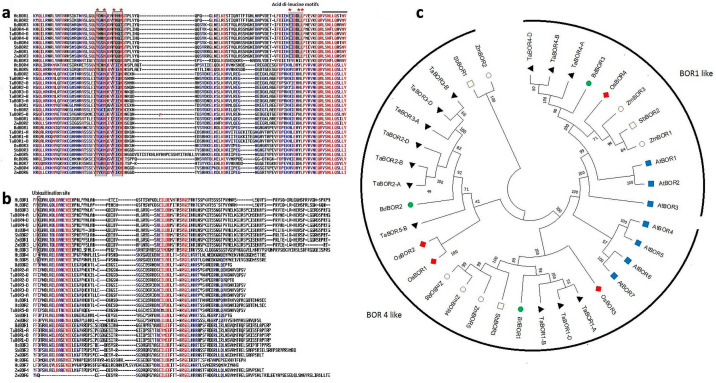
Analyses of multiple sequence alignments and phylogeny of BOR proteins. Highlighted motifs and regions are known to be essential for polarity and vacuolar sorting. (**a**,**b**) show important conserved regions present in BOR proteins. (**a**) Tyrosine-based motifs (YxxΦ), acidic di-leucine motifs ([D/E]xxxL[L/I]) and (**b**) ubiquitination site (K590) are found conserved in the studied BOR sequences. (**c**) shows the phylogenetic tree constructed using amino acid sequences of *A. thaliana*, *B. distachyon*, *O. sativa*, *S. bicolor*, *T. aestivum*, and *Z. mays* with MEGA X by the neighbor-joining method at the bootstrap value of 1000. The conventional division of B transporters into two groups i.e., BOR1-like and BOR4-like proteins, were observed. BOR4-like proteins were devoid of di-leucine motifs.

Multiple sequence alignment was performed to analyze the level of conservation among BOR proteins of selected cereals (Figure 2a,b and Figure S1). All the BOR-like sequences were found to be highly conserved. The sequences had some earlier reported motifs including “YxxM” and “FxxM” motifs [24] (Figure 2a). The conserved di-leucine motifs (E/DxxxLL/I) were exclusively present in AtBOR1, AtBOR2, TaBOR4-A, TaBOR4-B, TaBOR4-D, ZmBOR1, ZmBOR3, SbBOR2, and OsBOR4 (Figure 2a). Additionally, Lys^590^, which is also known to be a ubiquitination site, was conserved in these proteins along with AtBOR3 and AtBOR5 (Figure 2b).

Phylogenetic analysis of BOR proteins was carried out to understand their evolutionary relatedness. The phylogenetic tree remained tandem with the results of multiple sequence alignments. We could segregate the BOR proteins into two conventional groups, i.e., BOR1-like and BOR4-like proteins in the phylogenetic tree (Figure 2c). The identified homeologous and paralogous BORs were found to be tightly clustered in the same clade. Proteins including the TaBOR4 group, BdBOR3, OsBOR4, ZmBOR1, 3, SbBOR2 were present in the BOR1-like group, along with AtBOR1, AtBOR2 and AtBOR3 proteins. Proteins with di-leucine motifs and ubiquitination sites were exclusive to the BOR1-like group. All the other proteins, such as ZmBOR2, SbBOR1, TaBOR1 group, OsBOR2, and BdBOR1, were categorized in a BOR4-like group along with AtBOR4, AtBOR5, AtBOR6, and AtBOR7 of *A. thaliana* (Figure 2c).

### 2.3. Gene Structure Analysis

**Figure 3:**
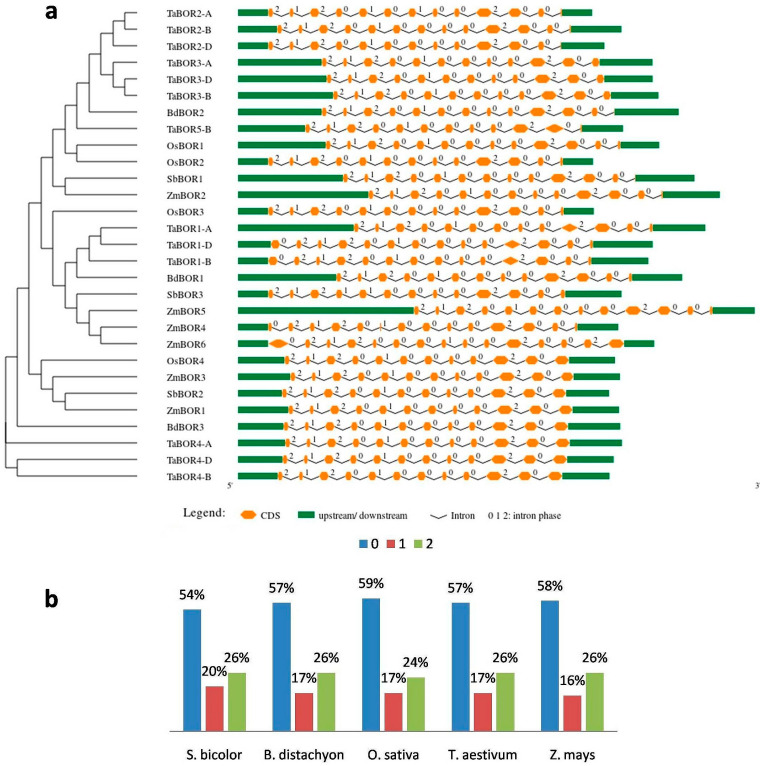
Gene architecture and intron phase distribution of *BOR* genes. (**a**) depicts the exon/intron organization of *BOR* genes in *B. distachyon*, *O. sativa*, *S. bicolor*, *T. aestivum*, and *Z. mays*. On average, around 13 exons were found in all five studied cereals. (**b**) shows the percentage distribution of intron phases in *BOR* genes. The majority of introns were found in phase 0, followed by phases 2 and 1.

The gene structure analysis provided insights into the architecture of *BOR* genes. The results were arranged according to the phylogenetic relationship among the *BOR* genes (Figure 3a). The average number of exons in *B. distachyon* and *S. bicolor* was 12.7 in both cereals, separately. *O. sativa* and *Z. mays* had 12.5 and 12.2 average exons, respectively. The highest numbers of exons were present in *T. aestivum*, with an average of 13.2. The maximum and minimum numbers of exons were 15 and 6 in *ZmBOR6* and *ZmBOR5*, respectively (Table S3). Intron analysis suggested that the majority of introns were present in phase 0, followed by phase 2 and phase 1, respectively. The average percentage shares of phases 0, 1, and 2 in all the cereals were 57%, 17%, and 26%, respectively, which again supported their conserved nature (Figure 3b).

### 2.4. Divergence Analysis

Divergence analysis was performed to find out various evolutionary aspects of the *BOR* genes. The values of Ka and Ks and their ratio were estimated for all three paralogous *TaBORs* (*TaBOR2-A-TaBOR3-D*, *TaBOR2-B-TaBOR3-B*, and *TaBOR2-D-TaBOR3-A*) to analyze the evolutionary discretion among the duplicated *TaBOR* genes. The negative Ka/Ks value of all three pairs, i.e., value < 1, suggested negative or purifying selection pressure during the evolution (Table 1). The range of divergence time of paralogous *TaBORs*, calculated using Ks value, was 23.7 to 25.2 million years ago (MYA) (Table 1). The Tajima’s relative rate test showed insignificant χ^2^ value for *TaBOR2-A-TaBOR3-*D, *TaBOR*2*-B-TaBOR3-B*, and *TaBOR2-D-TaBOR3-A* gene pairs at *p*-value > 0.05, thus following the molecular clock hypothesis, which states the equal rate of evolution among the gene pairs (Table 2) [25].

### 2.5. Protein Characterization

Protein characterization was performed to look more deeply into the various physicochemical aspects of BOR proteins (Table 3 and Table S3). The average lengths of BOR proteins of *B. distachyon*, *O. sativa*, *S. bicolor*, *T. aestivum*, and *Z. mays* were 683, 683, 684, 691, and 717 AA residues, respectively. The average MWs were 76.6, 76.7, 76.5, 77.2, and 80.2 kDa for BdBOR, OsBOR, SbBOR, TaBOR, and ZmBOR, respectively. Signal peptides were absent in the BOR proteins of all five cereals. The average pI values of both BdBOR and SbBOR, separately, were found to be eight, whereas 7.8, 7.6, and 8.2 were the average pI values for OsBOR, TaBOR, and ZmBOR proteins, respectively. The average GRAVY values of BdBOR, OsBOR, SbBOR, and TaBOR were found to be approximately 0.2 for each. However, the average GRAVY value for ZmBOR was 0.16. The bioinformatics’ tools, including CELLO, ngLOC, WoLFPSORT, and PROTCOMP, predicted the presence of all the BOR proteins in the plasma membrane (Table S4). In addition, ngLOC predicted localization of SbBOR2, BdBOR3, OsBOR4, ZmBOR1, ZmBOR3, and TaBOR4 in the plasma membrane or vacuole (Table S4).

A variety of tools were used to predict the transmembrane regions of BOR proteins. Around 9–12 transmembrane regions were predicted in BdBOR, OsBOR, and TaBOR proteins by Phobius and Protter; however, 8–10 and 5–12 TM regions were predicted in SbBORs and ZmBORs, respectively. TMHMM predicted 10–11 TM regions in BdBOR and TaBORs. Furthermore, 9–10, 9–11, and 5–10 TMs were predicted in OsBORs, SbBORs and ZmBORs, respectively. A minimum of 5 TMs were found in ZmBOR6, whereas a maximum of 12 TMs were predicted in BdBOR3, OsBOR3, ZmBOR1, TaBOR3-A, and TaBOR4 (Table 3 and Table S5). The majority of BOR proteins were bearing more than 10 transmembrane domains.

**Figure 4:**
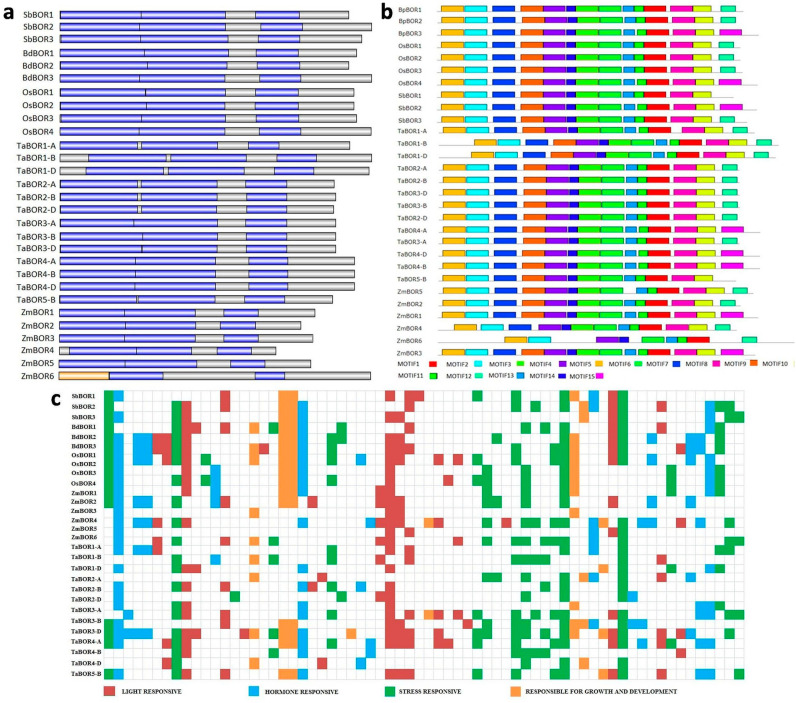
Domain, motif, and promoter analyses. (**a**) shows the domain distribution in the BOR proteins. HCO_3_^−^ domain (shown in blue color) was present in all of the BOR proteins. Orange color represents another domain, i.e., the ANTH domain, which was exclusively present in ZmBOR6 protein; (**b**) shows the 15 conserved motifs present in the BOR proteins of *B. distachyon*, *O. sativa*, *S. bicolor*, *T. aestivum*, and *Z. mays*; (**c**) shows the *cis*-regulatory elements present in the promoter regions of *BOR* genes of all five cereals. Brown, blue, green, and orange colors represent the light-responsive, hormone-responsive, stress-responsive, and growth- and development-related *cis*-regulated elements, respectively.

The domain architecture was explored to identify the domain structures present in BOR proteins. The domain analysis suggested the consistent presence of an anion exchanger bicarbonate (HCO_3_^−^) domain in all the identified BOR proteins (Table S6). Interestingly, ZmBOR6 had another domain, i.e., the ANTH domain in addition to the HCO_3_^−^ domain (Figure 4a). Furthermore, the MEME tool was used to analyze the 15 most conserved motifs in BOR proteins. A majority of the motifs were present in all the BOR proteins, including motifs 1, 2, 4, 5, 6, 11, 13, and 14. ZmBOR6 protein was void of motifs 3, 7, 8, 9, 10, and 15. Furthermore, motif 15 was found to be exclusive in SbBOR2, BdBOR3, OsBOR4, ZmBOR1, ZmBOR3, and TaBOR4 groups. Additionally, these proteins, along with SbBOR1, were found to be lacking motif 12 (Figure 4b).

### 2.6. Cis-Regulatory Elements

Promoter regions of *BORs* were analyzed for the presence of a diverse range of *cis*-regulatory elements. The regulatory elements were classified into four categories, including light-responsive, stress-responsive, hormone-responsive, and those involved in growth and development (Figure 4c and Table S7). AE-box, ATCT, BOX4, G-BOX, TCCC, as-1 motifs, etc., were some light-responsive elements. The stress-responsive elements found in *BORs* included A-box, ARE, LTR, MYB, MBS, STRE, WUN-motif, and W-box. Further, ABRE, AuxRR, ERE, GARE, P-box, TCA, etc., were found to be hormone-responsive elements. CAT box, CCGTCC, GCN4, RY element, O2-site, etc., were involved in the growth and development processes.

### 2.7. Expression Analysis of BOR Genes during Tissue Developmental Stages

**Figure 5:**
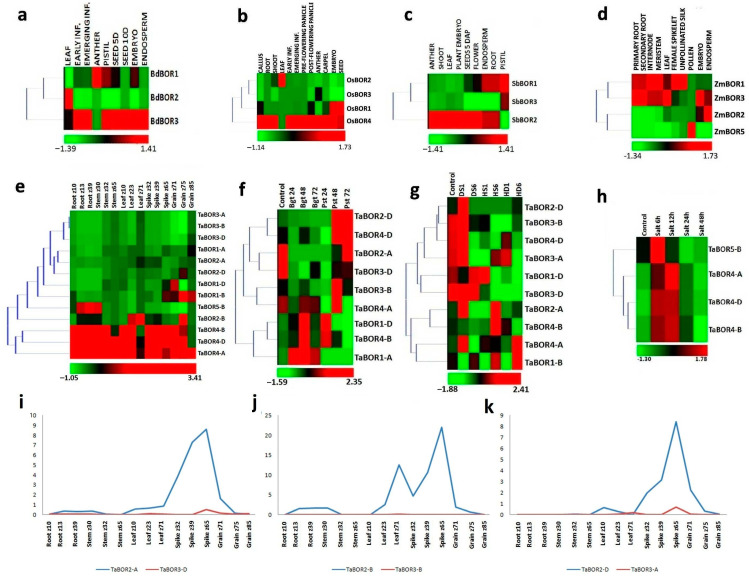
Expression analysis of *BOR* genes under various tissue developmental stages and abiotic and biotic stress conditions and comparative expression profiling of paralogous genes. (**a**–**e**) show the heat maps of *BOR* genes of *B. distachyon*, *O. sativa*, *S. bicolor*, *Z. mays*, and *T. aestivum*, respectively, during various tissue developmental stages. The expression of *BOR* genes of *T. aestivum* after the onset of Bgt and Pst infections is shown in the heatmap in (**f**). The expression profiling of *TaBOR* genes under heat, drought, and combined heat and drought stress are represented by (**g**). (**h**) shows the expression pattern of *TaBOR* genes during salt stress conditions. (**i**–**k**) represent the comparative expression trends of *TaBOR2-A-TaBOR3-D*, *TaBOR2-B-TaBOR3-B*, and *TaBOR2-D-TaBOR3-A*, respectively, under various tissue developmental stages of *T. aestivum*.

Expression profiling of *BOR* genes was carried out to identify their putative involvement in tissue development. Expression analysis in *B. distachyon* showed discrete expression during the development stages of plants. The highest expression was observed during anther and pistil development (Figure 5a). *BdBOR1* and *BdBOR3* showed varied expression during almost all the stages. The least expression was seen in *BdBOR2* during the tissue developmental stages of *B. distachyon.* The *BdBOR3* gene showed the highest expression in the anther tissue. In *O. sativa*, *OsBOR4* showed the highest expression in most of the tissue developmental stages. *OsBOR3* showed the least expression among them. Overall, the highest expression was observed by the majority of genes in the reproductive organ stages (Figure 5b). Expression analysis of *SbBORs* revealed the noticeable expression of these genes in the anther, pistil, and flower development stages. *SbBOR2* was the most-expressing gene during most of the tissue development stages (Figure 5c). The *Z. mays BOR* genes showed assorted expression during various developmental stages. *ZmBOR1*, *ZmBOR2, ZmBOR3*, and *ZmBOR5* showed their highest expression during the development of female spikelet, embryo, and root tissues, and pollen tissues, respectively (Figure 5d). In the case of *T. aestivum*, a majority of the *TaBORs* were found to be downregulated or remained neutral during tissue development stages (Figure 5e). However, *TaBOR4-A*, *B*, and *D* showed differential expression during the majority of the developmental stages, with the highest expression in spike tissue. In addition, *TaBOR5-B* showed modulated expression during the root (z13 and z39) and stem (z30) development stages. Interestingly, the *TaBOR1* and *TaBOR2* group genes were significantly upregulated in spike and grain tissues. Moreover, the expression patterns of duplicated pairs (*TaBOR2-A-TaBOR3-D*, *TaBOR2-B-TaBOR3-B*, and *TaBOR2-D-TaBOR3-A*) were also compared to decipher the functional similarities among them (Figure 5i–k). One of the genes in each paralogous pair was high-expressing in certain developmental stages, especially in spike tissue, while the other gene in each pair was very low-expressing. The results revealed pseudo-functionalization of duplicated genes during the course of evolution.

### 2.8. Expression Analysis of TaBORs in Different Stress Conditions

To understand the possible roles of *TaBOR* genes in combating different biotic and abiotic stresses, the expression profiling of these genes was carried out using high throughput RNA-seq data. In addition, a qRT-PCR experiment was also performed to find the roles of *TaBOR* genes under B stress conditions. Under biotic stresses, expression analysis after the infestation of Bgt showed significant expression of *TaBOR1-A* and *TaBOR1-D* during all the stages of infestation. Among them, the highest expression was observed after 48 h of Bgt stress conditions (Figure 5f). In addition, *TaBOR4-B* was moderately upregulated and *TaBOR2-D*, *TaBOR3-B*, and *TaBOR3-D* were downregulated after the infestation of Bgt. During Pst infestation, *TaBOR2-D* showed high expression after 48 h and 72 h. A moderate expression was observed in *TaBOR1-D*, *TaBOR3-B*, and *TaBOR4-B*, *D* during the Pst infestation. In DS conditions, *TaBOR2-A* and *TaBOR2-D* were starkly upregulated during the first hour of treatment. However, all other genes showed either moderated or downregulated expression. In HS treatments, *TaBOR2-A* and *TaBOR4-B* were upregulated after 6 h of treatment. *TaBOR1-B* showed moderate expression under the HD conditions (Figure 5g). Under 1M NaCl stress, only *TaBOR4-A*, *B*, *D* and *TaBOR5*-*B* genes were upregulated. These genes showed modulated expression during the initial stages, i.e., 6 h and 12 h of salt stress, which was gradually reduced in later stages (Figure 5h). The highest upregulation was observed in *TaBOR4-A* after 12 h of salt stress treatment. This pattern suggested the role of *TaBORs* in saline stress.

**Figure 6:**
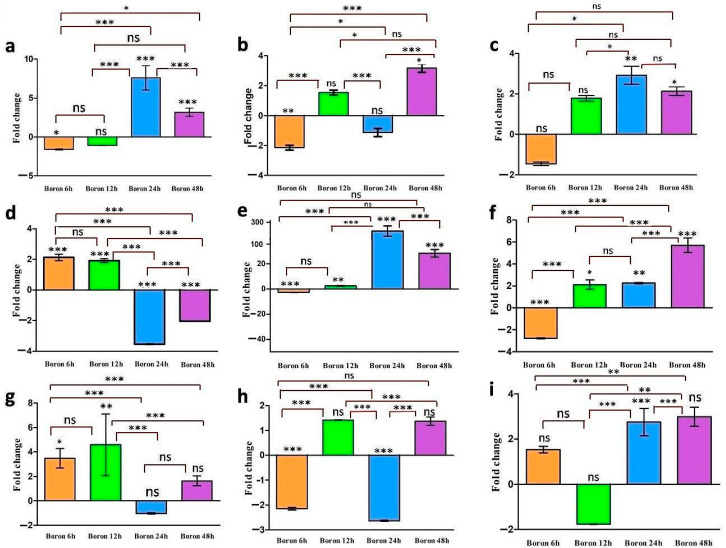
qRT-PCR analysis of five *TaBOR* genes. Five *TaBOR* genes were randomly selected for qRT PCR analysis. The expression pattern of *TaBOR1*-*B*, *TaBOR2-A*, *TaBOR3-D*, *TaBOR4-A*, and *TaBOR5-B* genes were analyzed under 20 mM B stress conditions. (**a**,**b**) show the expression of *TaBOR1-B* in roots and shoots, respectively. The expression pattern of *TaBOR2-A* in roots and shoots is shown in (**c**,**d**). (**e**,**f**) show the expression of *TaBOR3-D* in roots and shoots, respectively. *TaBOR4-A*’s expression pattern is depicted by (**g**,**h**) in roots and shoots, respectively. (**i**) shows the expression of *TaBOR5-B* gene in roots under B stress conditions. The analysis of variance (ANOVA) at 5% probability level was used to statistically analyze the data. The post hoc Tukey’s test was used to find the significant difference among the treatments (*p* < 0.05). *p*-value > 0.05, ≤0.05, ≤0.01, ≤0.001 is represented by symbols ns, *, **, and ***, respectively. The maroon-colored connecting lines show the significance among the treatments. GraphPad Prism 9 software was used to obtain graphs and statistical analyses for this data.

The qRT-PCR experiment was performed to analyze the expression of *TaBOR* genes under very high B stress (20 mM boric acid) conditions (Figure 6a–i). Representatives of all five groups of *TaBORs* were randomly selected and studied using gene-specific primers (Table S8). *TaBOR1-B* showed higher expression during the later stages of stress. Furthermore, maximum upregulation was observed after 48 h and 24 h in shoots and roots, respectively (Figure 6a,b). In *TaBOR2-A*, the expression in roots was gradually increased in the later stages of B stress (Figure 6c). In shoots, the gene was upregulated in the initial stages of stress and downregulated at 24 and 48 h (Figure 6d). In the *TaBOR3-D* gene, downregulation was seen in the initial stages of the expression and upregulation was observed in the later stages of B stress in both roots and shoots (Figure 6e,f). The highest expression was observed in the roots after 24 h B stress. In *TaBOR4-A*, maximum upregulation was observed at 12 h of B stress in both roots and shoots (Figure 6g,h). In *TaBOR5-B*, upregulation was evident throughout the B stress in roots with maximum expression at 48 h of stress (Figure 6i). The qRT data for shoots of *TaBOR5* was undetermined for all the B stress conditions, which might be due to their low expression in shoot tissues. The ANOVA test found significant changes between the treatment conditions in most of the genes.

### 2.9. Co-Expression Analysis

**Figure 7:**
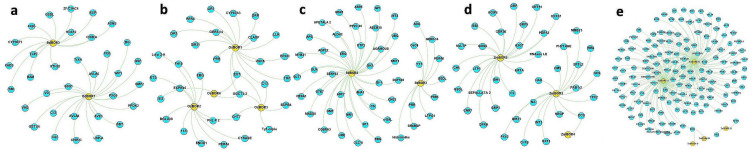
Co-expression analysis of *BOR* genes. (**a**–**e**) show the interaction network based on co-expression of *BOR* genes of (**a**) *B. distachyon*, (**b**) *O. sativa*, (**c**) *S. bicolor*, (**d**) *Z. mays*, and (**e**) *T. aestivum* during different tissue development stages.

To analyze the interaction of *BOR* genes with other genes of respective cereal, co-expression analysis was performed for each cereal (Figure 7a–h and Table S9). In *B. distachyon*, *BdBOR1* and *BdBOR3* were interacting with a total of 44 transcripts (Figure 7a and Table S9). For example, AVLA5 (avenin-like a5), PXG (probable peroxygenase 5), SBT (subtilisin-like protease), HOX12 (homeobox-leucine zipper HOX12), and TLP7 (thaumatin TLP7). Furthermore, the gene ontology mapping suggested their involvement in the discrete process; for instance, hydrolase activity (GO:0016788), glutathione transferase activity (GO:0004364), biotic stress-responsive activity (GO:0042742, GO:0050832), and nutrient reservoir activity (GO:0045735). A total of 67 transcripts of *O. sativa* genome were co-expressing with *OsBOR* genes (Figure 7b and Table S9). *OsBOR1* and *OsBOR2* were expressed with the majority of the transcripts, suggesting their diverse functions. As per gene ontology, these genes might also be involved in nutrient reservoir activity (GO:0045735), manganese ion-binding (GO:0030145), chitinase activity (GO:0004568), iron-binding (GO:0005506), nucleic acid-binding (GO:0003676), etc. In *S. bicolor*, *SbBOR2*, and *SbBOR3* interacted with 55 transcripts, including ERI2 (ERI1 exoribonuclease 2), DTF2 (factor of DNA methylation 2-like isoform X1), OMT (flavonoid O-methyltransferase), and LRR (leucine-rich repeat receptor kinase), and GSDL (GDSL esterase lipase), PRR4 (proline-rich 4), ADC (acyl-[acyl-carrier- ] desaturase chloroplastic-like), respectively (Figure 7c and Table S9). Gene ontology study suggested the roles of *SbBORs* in important cellular processes, including nitric oxide biosynthetic processes (GO:0006809), oxidoreductase activity (GO:0016491), fatty acid metabolic process (GO:0006631), and peroxidase activity (GO:0004601). In *Zea mays*, *ZmBOR6* interacted with a majority of transcripts encoding various important proteins, including HMA (heave metal-associated), CHT (chitinase), and DFRA (dihydroflavonol 4-reductase) (Figure 7d and Table S9). *ZmBORs* are predicted to be involved in peroxidase activity (GO:0004601), defense response (GO:0006952), lipid transport (GO:0006869), hydrolase activity (GO:0016787), protein phosphatase inhibitor activity (GO:0004864), oxidoreductase activity (GO:0016491), etc. by GO mapping. In the case of *T. aestivum*, *TaBOR* genes were co-expressing with 504 other genes of *T. aestivum* (Figure 7e and Table S9). Interestingly, group 1 *TaBOR* genes showed interaction with the majority of co-expressed genes. For instance, *TaBOR1*-*A* gene was expressed along with more than 200 other genes, such as *PLIM2B* (LIM domain-containing PLIM2b-like), *PMEI* (pectinesterase inhibitor-like), *CSLD3* (cellulose synthase D3), and RALF (rapid alkalinization factor-like). The *TaBOR2* and *TaBOR3* groups did not show the co-expressing trends with other genes. *TaBOR5-B* showed interaction with disease-resistance genes, including *RPM1* and *PIK2* genes (Figure 7e and Table S9).

## 3. Discussion

The BOR proteins were identified in *B. distachyon, O. sativa, S. bicolor, T. aestivum*, and *Z. mays* using extensive BLAST searches. All the identified proteins shared high homology with already-known seven BOR proteins (AtBORs) of *A. thaliana*, containing a conserved bicarbonate (HCO_3_^−^) domain [1,14]. In a previous study, 20 BOR transporters homologous to AtBORs were identified in *B. napus* [20]. Furthermore, a recent study identified a similar number of *BOR* genes in *B. distachyon*, *O. sativa*, and *S. bicolor* [26], whereas a higher number of *BOR* genes were identified in *Z. mays* in the present study. The highest number of genes belonged to the genome of *T. aestivum,* which could be due to its bigger genome size (~17 Gb) and the allohexaploid (AABBDD) nature of its genome [27]. The number of identified genes in some of the other cereals was found to be consistent with the earlier studies [1,20,22,28]. Furthermore, the analysis of paralogous genes suggested the exclusive occurrence of segmental DEs in *T. aestivum*. The segmental DEs are known to play important roles in the expansion of various gene families, which might also be responsible for the higher number of *BOR* genes in *T. aestivum* [29,30]. Further, the Ka/Ks analysis calculated a divergence time of 23.7 to 25.2 MYA among the paralogous *TaBOR* genes, suggesting the occurrence of DEs before the hybridization events of A B and D subgenomes [27]. The expression analyses of paralogous *TaBOR* genes revealed the negligible expression of one gene as compared to the other in each duplicated gene pair, suggesting pseudo-functionalization.

Multiple sequence alignments (MSA) provide deep insights into protein architecture. In our study, MSA suggested the conserved nature of BORs in various plant species. Myriad studies have shown the level of conservation in tandem with our study [9,20,24]. Most of the earlier reported conserved regions in these studies were present in the newly identified BOR proteins. For instance, Takano et al. showed that *A. thaliana* BOR1 is an efflux type B transporter for xylem loading [9]. In their study, they replaced two conserved amino acids (also found to be conserved in our sequences) S and G with P and E, respectively. The presence of the same amino acid residues highlights the trueness of the multiple sequence alignment and the conservational trend. Interestingly, tyrosine-based and phenylalanine-based motifs were also present in the identified BORs and are known to be responsible for signaling processes [24]. The presence of an acidic di-leucine motif in the same loop region as the tyrosine-based motifs in the BOR1-like proteins was also previously reported [24]. The di-leucine motif is responsible for polarity and B-dependent vacuolar sorting of AtBOR1 under low B conditions, which can be conserved in the clade; thus, it can be a possible function of other proteins present in the BOR1-like group [24]. The BOR1-like proteins had a ubiquitination site at K_590_ residue, which was reported to be essential for degradation and vacuolar sorting of AtBOR1 in response to high B supply [11].

Phylogenetic analysis was performed to explore the evolutionary relatedness of these proteins. The phylogenetic tree was categorized into two broad conventional groups (BOR1-like and BOR4-like) based on their similarity with AtBOR1 and AtBOR4 proteins, and earlier reported studies. The proteins present in the BOR-1 like group shared similarities, such as an exclusive presence of the di-leucine motif. A few of these proteins were characterized independently in earlier studies. For instance, TaBOR1.1, TaBOR1.2, and TaBOR1.3 are reported to be efflux type B-transporters, which were renamed as TaBOR4-A, TaBOR4-B, and TaBOR4-D in our study and belong to the BOR1-like group [18]. Furthermore, the OsBOR1 protein (OsBOR4 in our study) was present closer to the TaBOR1 (TaBOR4 in our study) and AtBOR1 and shared similar clade and functions [1,14,24]. Based on these analyses and phylogenetic clustering, we could predict the possible functions of other protein members of this clade, including BdBOR3, ZmBOR1, ZmBOR3, and SbBOR2, which might function as efflux type B-transporters. However, further validation of these results is required for a concrete statement. In the BOR4-like proteins, the OsBOR3 (OsBOR4 in previous studies) is known to be a pollen-specific B efflux transporter [19]. Two *O. sativa* proteins, OsBOR1 and OsBOR2, were present in the vicinity of the majority proteins of *T. aestivum* as well as BdBOR2 of *B. distachyon* (Figure 2c). These OsBOR1 and OsBOR2 are reported to be efflux type B-transporters [1,14,15,19]. Furthermore, Reid et al. (2007) found that the TaBOR2 (accession no. EU220225) was closer to the *O. sativa* BOR proteins of this clade and was found to be responsible for B efflux from root cells under high B conditions [15]. The other proteins of the same clade in our study might also have similar functions and can be studied further for more clarity.

The genome architecture was explored in terms of exons and introns. The exons and introns were distributed almost uniformly in all the identified *BOR* genes. It was seen that the variation among the introns indicates the evolutionary trend of the genes [31]. However, the uniformity of introns in BORs suggested the conserved nature among them during the evolutionary process. Further, the majority of introns were present in phase 0 in each cereal (Figure 3b), which also evinced the evolutionary conservation of *BOR* genes. Similar gene architecture of *BOR* genes has also been carried out in other plant species [20,21]. For instance, an average of 11.6 exons were present in *B. napus* [20]. Furthermore, 10 to 12 introns were found in 9 *BORs* of poplar [21]. These studies reflect the conservative nature of *BORs*.

Physicochemical characterization, including peptide length, MW, pI, GRAVY, subcellular location, and transmembrane regions, was performed to analyze various characteristic features of BOR proteins. Additionally, the domain architecture and motif distribution were studied. Similar genome-wide studies have been performed in various other plants, which suggest results consistent with our study. For instance, the lengths of *B. napus* BORs were in the range of 660 AA to 738 AA and MW ranging from 74.67 kDa to 81.24 kDa [20]. In *P. trichocarpa* BORs, protein sizes ranged from 666 AA to 731 AA, with an average MW of 77.8 [21]. Furthermore, the GRAVY value of identified BOR proteins suggested the hydrophilic property of BOR proteins; however, a negative GRAVY value of ZmBOR6 suggested its possible hydrophobic nature. Except for ZmBOR6, all other BOR proteins had GRAVY values similar to those of BnBOR proteins [20]. The disparity might be attributed to the difference in the architecture of ZmBOR6 from all the other members.

The presence of BOR proteins in the plasma membrane was earlier reported in numerous studies [1,14,18]. Leaungthitikanchana et al. (2013) reported the presence of TaBOR1 proteins in the plasma membrane [18]. The *SiBOR1* of *Setaria italica* was also found to encode a plasma membrane protein [23]. In the case of *B. napus*, the in silico tool WoLF PSORT suggested the localization of BORs in the plasma membrane, similar to our results. We used various tools to predict the localization of BOR proteins; a majority of them suggested the presence of BOR proteins in the plasma membrane. The membrane localization of these proteins further supports their role as transporters. Furthermore, in our study we found the similar pattern of the presence of transmembrane (TM) domains, as compared with different relevant studies. For instance, a majority of BORs in our study had more than 10 TMs. In *P. trichocarpa*, the number of TM domains was in the range of 10–12 [21]. AtBOR1 and OsBOR1 had 10 TM domains [14]. The domain architecture of BOR proteins revealed the similarity in the domain distribution among BORs. The signature HCO_3_^−^ domain was uniformly distributed in all the studied BOR proteins. ZmBOR6, however, showed a distinct pattern, as it had an additional ANTH domain. This protein might be involved in pollen germination processes, as the ANTH domain is associated with pollen germination [32]. Motif analysis was performed to study the functional similarity pattern of *BOR* genes. The presence of common motifs in most of the BOR proteins of different plant species supports their functional conservation. In a previous study, Ozyigit et al. (2020) identified the five most conserved motifs in 18 different plant species. They suggested that these motifs can also be used as a signature in identifying BOR proteins in unidentified plant species [26]. Overall, the protein characterization study provided us an opportunity to examine the disparities and similarities among various characteristic features of these proteins.

The promoter region of each *BOR* was analyzed for the presence of *cis*-regulatory elements to obtain hints about their possible involvement in different processes. Several light-responsive, stress-responsive, hormone-responsive, and growth- and development-responsive elements were identified. The results suggested the assorted roles of *BOR* genes in various regulatory phenomena of plants. In a similar study, Chen et al. identified some *cis*-regulatory elements involved in the regulation of *BOR* genes in *B. napus* such as GATA and I-box. [20]. Furthermore, in a study on *B. napus*, WRKY transcription factors that bind to W-box were reported to be involved in response to B stress conditions [33].

The expression analyses of *BOR* genes were performed in tissue developmental stages and different biotic and abiotic stress conditions. The majority of these genes showed perceptible expression in various developmental stages, especially in reproductive organ development. The higher expression of *BOR* genes of all cereals in reproductive organs suggested their key roles in plant reproduction. In 2013, Tanaka et al. reported that the *OsBOR4* was highly expressed in floral tissues. In another study, the *BOR* gene was reported to be involved in the developmental process of grapes [16]. Moreover, *SiBOR1* was reported to be involved in the developmental processes of foxtail millet [23]. Furthermore, in our study, numerous genes, including *OsBOR4*, *SbBOR2*, *TaBOR4* group, *TaBOR5-B*, *ZmBOR1*, and *ZmBOR3*, showed modulated expression in the roots; those genes are probably involved in B homeostasis in the root tissue. In a similar report, Chen et al. (2018) suggested the expression of *BOR* genes of *B. napus* in the root tissue [20]. Ostensibly, *BOR* genes might play roles in the development of reproductive tissues in almost all these studied plant species, which can be further validated in future studies. Expression trends of the paralogous gene pairs were also compared and analyzed in various tissue and development stages. All three pairs showed pseudo-functionalization. The nature of pseudo-functionalization rather than of neo-functionalization suggests their conserved function; however, that needs to be validated in future studies.

The expression profiling of *TaBOR* genes under different biotic and abiotic stress conditions provided important results. The modulated expression of a few *TaBOR* genes during fungal (Bgt and Pst) infestation and various abiotic stresses (HS, DS, HD, and salt) indicated their role in these stress conditions. Hitherto, we could not find any similar study that examined these aspects of *BOR* genes. Most of the studies were confined to exploring the expressional trends under B stress conditions. However, our study provides additional insight into their role in combating these biotic and abiotic stress conditions.

To study their differential expression pattern in boron stress, a qRT-PCR experiment was performed for five randomly selected *TaBOR* genes. The results provided intriguing patterns of expression of these genes during high B stress conditions. In an earlier study, Hua et al. (2017) reported the upregulation and downregulation of *BOR* genes under toxicity, as well as the deficiency of B in *B. napus*. The overall expression profiling revealed that *BnaBORs* were preferentially expressed in the roots of *B. napus* [34]. In another study, a research group performed a qRT-PCR analysis of *TaBOR4-A*, *TaBOR4-B*, and *TaBOR4-D* (named TaBOR1.1, TaBOR1.2, and TaBOR1.3, respectively, in the earlier study) under 1 mM B conditions [18]. The comparative study suggested the highest expression of *TaBOR4-B* under excess stress, albeit the expressions of the other two genes were perceptible. In our study, the *TaBOR4-A* gene was explored. It showed initial expression under very high B treatment, thereby indicating its possible role in combating B stress conditions. In another study, Nakagawa et al. (2007) reported the significant upregulation of *OsBOR1*, closest to the *TaBOR5-B* in the evolutionary tree, in roots [14]. In our analysis, it was observed that *TaBOR5-B* showed significant expression in roots. The results indicated that some of these genes are directly associated with the B homeostasis, which needs to be further validated individually in subsequent studies.

The co-expression analysis of *BOR* genes in each cereal was performed. In earlier studies, co-expression analysis has been used to determine brief indications about the involvement of genes in a variety of functions [35]. The interaction of *BOR* genes with other genes, such as *LRRK*, *CPK*, *ACR*, *STY*, and other kinases, suggests their roles in signaling processes. Furthermore, the genes interacting with *TLP*, *LEA*, *chitinases,* etc., indicate their involvement in the defense mechanisms. Moreover, the interaction of genes, such as peroxidases and ascorbate oxidases, evinces their roles in antioxidation pathways. The interactions of *BORs* with expansin genes indicate their involvement in cell-wall synthesis and integrity. Regulation of various hydrolytic enzymes and antioxidants was also reported earlier in the *BOR* genes of *B. napus* [34]. In addition, various co-expressing genes hint at the numerous indirect roles of *BOR* genes in regulations of different processes, and growth and development. The co-expression findings were also well supported by the analysis of *cis*-regulatory elements present in the *BOR* genes, where several elements important for hormone-responsive, stress-responsive, light-responsive, and growth- and development-responsive processes were discussed. However, the diverse roles of *BOR* genes should be validated individually in future studies.

## 4. Materials and Methods

### 4.1. Identification and Nomenclature of BOR Genes

The identification of BOR proteins in the genomes of *B. distachyon*, *O. sativa*, *S. bicolor, T. aestivum*, and *Z. mays* was done using standard methods [35,36]. BLASTp searches at e-value e^−10^ were performed against the genome of each cereal, separately, using known *A. thaliana* BOR sequences as the query sequences at the Ensembl Plants portal (https://plants.ensembl.org, accessed on 21 May 2020) [37]. The sequences with ≥90% query cover with significant similarity were considered for further study. HMMER and the Pfam BLAST searches [38] were performed to detect the presence of the conserved anion exchanger bicarbonate (HCO_3_^−^) domain (PF00955). An NCBI conserved domain database BLAST search was also performed. All the *BOR* genes were named according to their chromosomal distribution except *T. aestivum*, the nomenclature of which was done according to standard international rules (http://wheat.pw.usda.gov/ggpages/wgc/98/Intro.html, accessed on 7 July 2020).

### 4.2. Chromosomal Mapping and Duplication Events

The chromosomal and sub-genomic positions of the genes of each cereal were retrieved from the Ensembl Plants portal. The identification of homeologous *TaBOR* genes was performed using the bidirectional BLAST search (e-value 10^−10^) with a sequence similarity of ≥90% and on the basis of localization on the homeologous chromosomes group of A, B, and D subgenomes [39]. The identified *BOR* genes were mapped to their respective chromosomes using MapInspect (http://www.plantbreeding.wur.nl/uk/software_mapinspect.html.2012, accessed on 30 June 2020). Duplication events (DEs) were identified using the bidirectional BLAST hit approach with sequence similarity ≥80%. The differentiation of segmental and tandem DEs was carried out on the basis of distance [40,41].

### 4.3. Multiple Sequence Alignment and Evolutionary Analysis

Multiple sequence alignment was performed using the MultAlin (http://multalin.toulouse.inra.fr/multalin/, accessed on 2 September 2020) and MUSCLE programs [42,43]. Full-length BOR protein sequences of *A. thaliana, B. distachyon, O. sativa, S. bicolor, T. aestivum*, and *Z. mays* were used for the construction of the phylogenetic tree. The MUSCLE program was used for the sequence alignment and an evolutionary tree was constructed using the MEGA X program with the neighbor-joining (NJ) method at the bootstrap value of 1000 [44].

### 4.4. Ka/Ks Substitutions

The ClustalOmega server was used to align protein and nucleotide sequences of duplicated genes [45]. Synonymous substitution per synonymous site (Ks) and non-synonymous substitution per non-synonymous site (Ka) and their ratio, i.e., Ka/Ks, were calculated using the PAL2NAL server [46,47]. The divergence time was calculated using the formula “T = Ks/2r,” where T represents the divergence time and r represents the divergence rate, which was assumed to be 6.5 × 10^−9^ for cereals [48].

### 4.5. Gene Structure Analysis

The genomic and coding sequences were aligned and analyzed for the exon-intron organization of the putative *BOR* genes. The number of exons and introns were retrieved from the Ensembl Plants portal for these genes. Pictorial representation was developed using the Gene Structure Display Server (GSDS 2.0) [49].

### 4.6. Promoter Analysis

An upstream region of around 1500 bp of each *BOR* gene was analyzed for the presence of various *cis*-regulatory elements. The PlantCARE [50] server was used to determine the different types of regulatory elements.

### 4.7. Protein Characterization

The Expasy tool and sequence manipulation suite (SMS) were used to compute the theoretical molecular weight (MW), isoelectric point (pI), and GRAVY value of identified BOR proteins of five cereals [51,52]. Transmembrane (TM) regions were predicted using Phobius, Protter, and TMHMM v.2.0 [53,54,55]. Subcellular localization was predicted using CELLO, WoLF PSORT, PROTCOMP, and ngLOC [56,57,58,59]. The SignalP was used for the prediction of signal peptides [60]. The SMART server was used for domain analysis, and the IBS server was used for pictorial representation [61]. Motifs of BOR proteins were computed using the Maximization for Motif Elicitation (MEME) version 4.11.4 [62].

### 4.8. Expression Profiling Studies

High throughput RNA-seq data were retrieved from the URGI database (urgi.versailles.inra.fr/files/RNASeqWheat/, 25 February 2019) and Expression ATLAS [63,64,65] for expression analysis. For *T. aestivum,* the expression data generated in replicates for various tissue development stages and under various abiotic and biotic stresses were used [66,67,68]. To analyze the tissue-specific expression in wheat, high throughput RNA-seq data (ERP004714) for the three developmental stages of root, stem, leaf, spike and grain tissues were used. RNA-seq data in duplicates (SRP045409) for one and six hours (h) treatments of heat (40 °C), drought (20% PEG 6000) and the combination of both heat and drought stresses were used for the analysis of *TaBOR* genes under abiotic stress conditions [67]. RNA-seq data for roots generated by Zhang et al. in triplicate for salt stress at 6, 12, 24, and 48 h were used for expression profiling of *TaBOR* genes under saline stress [68]. For expression analysis of *TaBORs* under biotic stresses, triplicate RNA-seq data (PRJNA243835) after the inoculation of *Blumeria graminis f. sp. tritici* (Bgt) and *Puccinia striiformis f. sp. tritici* (Pst) for one, two, and three days in seven-day-old seedlings were used [67]. The WheatExp server was used to validate the RNA-seq datasets [69]. Expression value was calculated in terms of FPKM value using the Trinity package [70]. Genes having fold values >2 in any of the stages were included for further analysis. Heat maps of expressed genes of cereals were generated using Hierarchical Clustering Explorer 3.5, clustered with the Euclidean distance method [71].

### 4.9. Co-Expression Analysis

Co-expression analysis of *BOR* genes was performed using the whole expression data of each cereal. CoExpress v.1.5 was used for co-expression analysis with the threshold value ≥ 0.9, correlation power 1 with 100 bootstraps [72]. Functional annotations of co-expressed *BOR* genes were carried out using Blast2GO [73]. Gephi 0.9.1 was used for the pictorial representation of interacting genes [74].

### 4.10. Plant Growth, Treatments, and qRT-PCR Experiment

*T. aestivum* (cv. Chinese spring) seeds were surface-sterilized using sodium hypochlorite (1.2%) in 10% ethanol. Sterilized seeds were washed with double autoclaved water and germinated on moist Whatman filter papers for 3–5 days. After transferring germinated seedlings to fresh phytaboxes, they were grown in a plant growth chamber. Seven-day-old seedlings were subjected to B stress (20 mM) for 6, 12, 24, and 48 h, separately. Seedlings grown in normal conditions were used as a control. The samples (roots and shoots) were collected, frozen in liquid nitrogen, and stored at −80 °C until further use. The samples were used for the isolation of total RNA using the Spectrum^TM^ Plant Total RNA Kit (Sigma, Saint Louis, MI, USA). The contamination of genomic DNA was removed using the TURBO DNA-free™ Kit (Invitrogen, Carlsbad, CA, USA). Agarose gel electrophoresis and nanodrop quantification were used for qualitative and quantitative analysis of RNA, respectively. The cDNA was synthesized with one microgram of total RNA using the Superscript III First-Strand Synthesis Super-mix (Invitrogen, Carlsbad, CA, USA). Gene-specific primers of selected genes were used for the qRT-PCR experiment that was performed using the 7900 HT Fast Real-Time PCR System (Applied Biosystems) with SYBR Green [29]. The results were analyzed in the form of fold expression change by the delta-delta CT method (2^−ΔΔCT^) [75]. The expression of the ADP-ribosylation factor (*TaARF1*) was used as an internal control [76,77]. All the experiments were performed in three biological replicates and expressed as mean ± SD. The analysis of variance (ANOVA) at the 5% probability level was used to statistically analyze the data. A post hoc Tukey’s test was used to find the significant difference among the treatments (*p* < 0.05).

## 5. Conclusions

B is an essential micronutrient for plants. However, both the toxicity and the deficiency of B may lead to complications for the growth and development of plants. BOR transporters are specialized proteins entailed in the B uptake and homeostasis. The present study vets the BOR transporters present in the genome of five cereals, including *B. distachyon*, *O. sativa, S. bicolor*, *T. aestivum*, and *Z. mays*. A total of 29 genes identified in the studied cereals had a common bicarbonate domain (HCO_3_^−^), which acted as a signature domain. Further, multiple sequence alignments provided a terse indication of high conservation among the BOR proteins. Evolutionary analyses indicated the role of DEs and purifying selection in the expansion of BOR gene family. Moreover, gene structure, protein structure, domain, and motif analyses showed the conserved architecture of BORs. To foresee functional aspects of *BOR* genes, several investigations, including *cis*-regulatory elements analysis, expression analysis, and co-expression analysis using high throughput RNA-seq data, were performed. These analyses suggested the possible role of *BOR* genes in different growth and developmental processes and stress-responsive activities apart from the B homeostasis. The qRT-PCR experiment performed under B-treated *T. aestivum* plants ascertained the involvement of the *TaBOR* genes in response to B stress conditions. However, further research is required to look more deeply into the *BOR* genes. The study will be ancillary for research into the *BOR* genes in different plants. The results will be helpful in various crop improvement programs.

## Figures and Tables

**Table 1:** The Ka/Ks ratio and divergence time of *TaBOR* paralogous genes.

Gene A	Gene B	Ka	Ks	Ka/Ks	Selection Pressure	T = Ks/2r
*TaBOR2-A*	*TaBOR3-D*	0.0581	0.3206	0.1814	Purifying	24.7
*TaBOR2-B*	*TaBOR3-B*	0.0570	0.3275	0.1741	Purifying	25.2
*TaBOR2-D*	*TaBOR3-A*	0.0614	0.3081	0.1992	Purifying	23.7

Ka: non-synonymous substitutions per non-synonymous site; Ks: synonymous substitutions per synonymous site; T: divergence time.

**Table 2:** Tajima’s relative rate test of duplicated gene pairs.

Group A	Group B	Out Group	Nt	Na	Nb	χ^2^	P
*TaBOR2-A*	*TaBOR3-D*	*TaBOR4-A*	1149	64	83	2.46	0.11709
*TaBOR2-B*	*TaBOR3-B*	*TaBOR4-A*	1156	79	77	0.03	0.87278
*TaBOR2-D*	*TaBOR3-A*	*TaBOR4-A*	1145	73	77	0.11	0.74397

Nt: identical sites in all three sequences; Na: unique differences in Sequence A; Nb: unique differences in Sequence B.

**Table 3:** Characteristic features of BOR proteins of *B. distachyon*, *O. sativa*, *S. bicolor*, *T. aestivum*, and *Z. mays*.

	*B. distachyon*	*O. sativa*	*S. bicolor*	*T. aestivum*	*Z. mays*
Average length (aa)	683	683	684	691	717
Average MW (kDa)	76.6	76.7	76.5	77.2	80.2
Signal peptides	0	0	0	0	0
Average pI value	8	7.8	8	7.6	8.2
Average GRAVY	0.2	0.2	0.2	0.2	0.16
Sub-cellular localization	PM	PM	PM	PM	PM
Transmemberane regions (TMs)	9–12	9–12	8–10	9–12	5–12

## Data Availability

All the data used in this study are freely available in the databases /repositories. Link and accession numbers have been mentioned in the manuscript.

## Supplementary Materials

The following supporting information can be downloaded at: https://www.mdpi.com/article/10.3390/plants11070911/s1, Table S1: Nomenclature of *BOR* genes in *B. distachyon*, *O. sativa*, *S. bicolor*, *T. aestivum*, and *Z. mays*. Table S2: List of paralogous *TaBOR* genes. Table S3: Characterization of *BOR* genes and proteins in *B. distachyon*, *O. sativa*, *S. bicolor*, *T. aestivum*, and *Z. mays*. Table S4: Subcellular localization of BORs of *B. distachyon*, *O. sativa*, *S. bicolor*, *T. aestivum*, and *Z. mays* predicted by various tools. Table S5: Transmembrane regions of BORs of *B. distachyon*, *O. sativa*, *S. bicolor*, *T. aestivum*, and *Z. mays* predicted by different tools. Table S6: Domain analysis of BOR proteins of *B. distachyon*, *O. sativa*, *S. bicolor*, *T. aestivum*, and *Z. mays*. Table S7: Cis-regulatory elements in *S. bicolor*, *B. distachyon*, *O. sativa, Z. mays*, and *T. aestivum*, respectively. Brown, blue, green, and orange colors represent the light-responsive, hormone-responsive, stress-responsive and growth- and development-related cis-regulated elements, respectively. Table S8: qRT-PCR primers of *TaBOR* genes and *TaARF* gene. Table S9: Annotation and abbreviation of co-expressed genes of *T. aestivum* during various tissue developmental stages. Figure S1: Multiple sequence alignments of BOR proteins of *B. distachyon*, *O. sativa*, *S. bicolor*, *T. aestivum*, and *Z. mays*.
