# Supplementary figures and images for: Molecular Characterization, Evolutionary Analysis, and Expression Profiling of BOR Genes in Important Cereals
Source: Plants (Basel). 2022 Mar 29;11(7):911. doi: 10.3390/plants11070911 (PMC9002812; doi:10.3390/plants11070911)

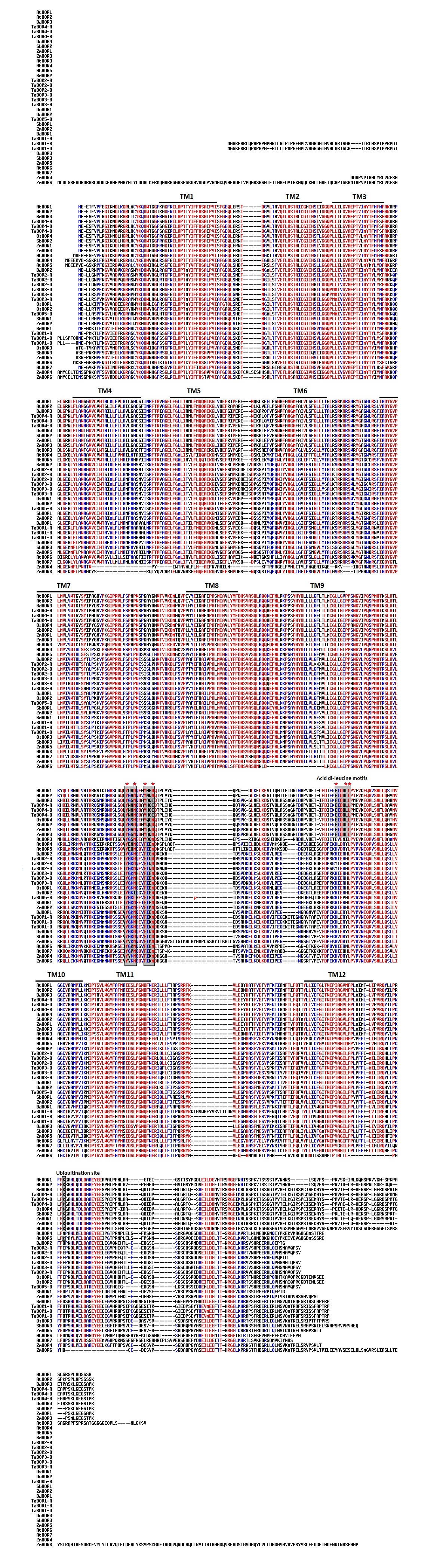

Supplement: Supplementary file 1 [file plants-11-00911-s001.zip › Supl. Files/Figure S1.jpg]
